# Knowledge mobilisation for policy development: implementing systems approaches through participatory dynamic simulation modelling

**DOI:** 10.1186/s12961-017-0245-1

**Published:** 2017-10-02

**Authors:** Louise Freebairn, Lucie Rychetnik, Jo-An Atkinson, Paul Kelly, Geoff McDonnell, Nick Roberts, Christine Whittall, Sally Redman

**Affiliations:** 1 ACT Government, Health Directorate, GPO Box 825, Canberra, ACT 2601 Australia; 2 The Australian Prevention Partnership Centre, Sax Institute, PO Box K617, Haymarket, NSW 1240 Sydney, Australia; 3 School of Medicine, University of Notre Dame, PO Box 944, Broadway, NSW 2007 Sydney, Australia; 4 Sydney Medical School, University of Sydney, Sydney, NSW 2006 Australia; 5 The Australian National University, Canberra, ACT 2601 Australia; 6 Adaptive Care Systems, Sydney, NSW 2052 Australia; 7 NSW Ministry of Health, LMB 961 North, Sydney, NSW 2059 Australia

**Keywords:** Participatory dynamic simulation modelling, Decision support, Knowledge mobilisation, Childhood obesity, Alcohol, Diabetes in pregnancy

## Abstract

**Background:**

Evidence-based decision-making is an important foundation for health policy and service planning decisions, yet there remain challenges in ensuring that the many forms of available evidence are considered when decisions are being made. Mobilising knowledge for policy and practice is an emergent process, and one that is highly relational, often messy and profoundly context dependent. Systems approaches, such as dynamic simulation modelling can be used to examine both complex health issues and the context in which they are embedded, and to develop decision support tools.

**Objective:**

This paper reports on the novel use of participatory simulation modelling as a knowledge mobilisation tool in Australian real-world policy settings. We describe how this approach combined systems science methodology and some of the core elements of knowledge mobilisation best practice. We describe the strategies adopted in three case studies to address both technical and socio-political issues, and compile the experiential lessons derived. Finally, we consider the implications of these knowledge mobilisation case studies and provide evidence for the feasibility of this approach in policy development settings.

**Conclusion:**

Participatory dynamic simulation modelling builds on contemporary knowledge mobilisation approaches for health stakeholders to collaborate and explore policy and health service scenarios for priority public health topics. The participatory methods place the decision-maker at the centre of the process and embed deliberative methods and co-production of knowledge. The simulation models function as health policy and programme dynamic decision support tools that integrate diverse forms of evidence, including research evidence, expert knowledge and localised contextual information. Further research is underway to determine the impact of these methods on health service decision-making.

## Key messages

- Participatory dynamic simulation modelling is being implemented as a knowledge mobilisation strategy in Australian health policy settings.
- Key elements of this knowledge mobilisation approach has included:
  - Moving beyond evidence synthesis to providing dynamic decision support tool to compare policy options.
  - Embedding deliberative methods to build shared understanding of complex issues and intervention outcomes.
  - Emphasising stakeholder participation in the co-production of knowledge.
- Operationalising participatory simulation modelling relied on:
  - Effective and equal partnerships.
  - Active participation of all stakeholders in the modelling process.
  - Transparency and trust in model outputs to facilitate consensus for action.

## Background

The utilisation of evidence has come a long way since the advent of evidence-based medicine – a term coined in the early 1990’s [1–3] when the leading proponents were described as radicals [4]. Evidence-based healthcare captured the zeitgeist and coalesced into a mainstream movement built on decades of population-based research, clinical epidemiology, critical appraisal and systematic review methods [5]. Interventions aimed at supporting the use of evidence in policy and practice have spawned new theories and frameworks, translation research, and an evolving lexicon [6]. A recent scoping review identified 51 different taxonomies to categorise research translation interventions [7].

Yet, despite great progress and mainstream acceptance of evidence-informed decision-making there remain many operational challenges. Researchers who understand the scientific evidence are often not engaged, or unheard, when important policy decisions are made [8, 9]. Similarly, practitioners familiar with the local context and those who are considered the ‘end users’ of the research are often not engaged in the research process [10]. A common dilemma is the apparent mismatch between the information priorities of policy decision-makers and programme or service funders, and the research priorities of investigators and research funders [11]. To be policy relevant, research must reflect an understanding of decision-making environments, be responsive to end-user needs, and be supported with stakeholder engagement and strategic communication [11, 12]. Contemporary thinking also suggests locally co-created knowledge, derived from researcher and end-user partnerships, preferably on the location where it is to be applied, is particularly useful for addressing policy and practice questions [13, 14]. This is akin to Senge’s description of the art and practice of collective learning [15].

The conceptual and empirical developments of contemporary strategies to support evidence-informed action are reflected in the evolving terminology. While knowledge translation focuses on the transfer of investigator-driven research to policy and practice settings [6, 11, 16], knowledge exchange has emphasised the relational two-way communication required for research uptake to be effective and useful [17, 18]. The most recent adoption of knowledge mobilisation further highlights organisational structures and system design requirements, and more explicitly values the ‘co-creation’ of knowledge [19, 20]. It is also the broadest term to encompass all activities that involve generating, sharing and using research [19]. Best et al. [21, 22] described these developments as the three generations of translation, namely linear, relational and systems-based approaches. Whichever terminology one may prefer, however, it is widely acknowledged that mobilising knowledge for policy and practice is an emergent process, and one that is highly relational, often messy and profoundly context dependent [23–27].

Systems thinking and systems science have growing influence on many aspects of public health discourse and research [28, 29]. Important elements of systems thinking include more conscious attention to how new forms of knowledge are “*gained, managed, exchanged, interpreted, integrated and disseminated*”, and an emphasis on “*transdisciplinary, translational and network-centric*” science [28]. There are natural synergies between knowledge mobilisation methods and systems science methods. Knowledge mobilisation refers to a range of active approaches deployed to encourage the creation and sharing of research-informed knowledge [30]. Systems science methods encompass a family of approaches that can be used to elucidate the behaviour of complex systems, inform efforts to address one or more system problems [31], and have the capacity to examine both complex health problems and the context in which they are embedded [29, 32, 33]. Key elements of a systems science approach include synthesising diverse knowledge and evidence, exploring the potential for non-linear relationships between contributing factors and unanticipated emergent behaviour of the complex systems (including policy resistance) [31, 34, 35]. The value of systems thinking for conducting reviews of evidence and integrating other forms of knowledge are well described [26, 36]. However, applying systems thinking to knowledge mobilisation is conceptually challenging and difficult to operationalise [37]. A recent multi-method review of knowledge mobilisation across health and other sectors concludes that the most fruitful lessons about the future role of systems thinking will come from natural experiments and case studies [19].

In this paper, we describe our experience of implementing a systems-based approach of participatory dynamic simulation modelling as a knowledge mobilisation strategy in Australian real-world policy settings. We describe how this approach combined both systems science methodology and some of the core elements of knowledge mobilisation best practice using three case studies (two published [38, 39] and one as yet unpublished). We describe the strategies adopted to address both technical issues (e.g. synthesising diverse evidence into a quantifiable model) and socio-political issues (e.g. user engagement and trust), and compile the experiential lessons derived. Finally, we consider the implications of these knowledge mobilisation case-studies and provide evidence for the feasibility of this approach in policy development settings.

## Participatory dynamic simulation modelling draws on many elements of knowledge mobilisation best practice

Dynamic simulation modelling is a systems science method that recreates complex systems and human behaviours in a virtual world. These models can answer ‘what if’ questions about the likely impacts over time of different policy and intervention options and combinations so that they can then be deliberated and considered more broadly before implementation in the real world [40, 41]. Dynamic simulation modelling has been used to map health system components and their interactions, synthesise evidence, examine and compare the potential outcomes of interventions, and guide more efficient investment and conscientious disinvestment of resources [41]. This is important for preventive health policy and practice, where decision support tools must have the capacity to steer a course through the complexity of interactions that give rise to real-world public health problems such as the global epidemic of chronic disease [40–42].

The concept of ‘evidence-informed decisions’ is challenging in population health policy and practice interventions that require engagement and partnership with sectors outside of health. Many factors, including types of information, opinion and experience, timing, the political cycle, local norms, the influence of external players, and the availability of funds, all influence decision-making [9, 43]. Many of the current ‘big questions’ in public health are complex and not easy to address. These problems have multiple interacting causal factors with competing possible courses of action for decision-makers to choose between, each course of action potentially resulting in complex and unintended consequences [40, 44].

However, to date, the potential of participatory simulation modelling as a knowledge mobilisation tool in the health sector has not been adequately explored. In particular, stakeholder engagement and involvement of end-users in health-related simulation model development has been lacking [41]. This has limited the use of simulation modelling across the range of potential applications, hindered the implementation of model findings [45, 46] and led to a reluctance among ‘non-researchers’ to use models as decision support tools [46, 47]. A systematic review of the use of simulation modelling to inform surgical patient flow processes found that only half of publications stated that the goal of the model was to inform policymakers and health service managers, and only 26% actually included these end-users in the simulation modelling process [10].

Below, we discuss how participatory simulation modelling can build on contemporary knowledge mobilisation approaches to offer a tool for timely and dynamic policy decision support, both by embedding deliberative methods and emphasising the co-production of knowledge in the modelling process. We then reflect on the experience and learnings drawn from three Australian case studies of participatory dynamic simulation modelling conducted in collaboration with jurisdictional health departments [38, 39].

### Moving from evidence synthesis to timely and dynamic decision support

Evidence-informed policy and practice has traditionally relied on systematic reviews, evidence summaries and policy briefs to provide decision-makers with rigorous, timely and concise information [48–50]. While their inherent value is acknowledged, there are limitations in their use and utility for health policy decision-making [12].

Systematic reviews and meta-analyses synthesise the available evidence to answer the question ‘what do we know about this issue?’ They focus on clear and specific questions and usually have a narrow scope of investigation with limited potential to examine complex questions [51, 52]. These methods produce static reports that rely on decision-makers to navigate the complexity and uncertainty of translating the evidence for their local context and weigh up the options for responding to their problem [53]. Many systematic reviews fail to address the policy implications of their findings [12] in a timely way to inform decision-making [54].

More recently, there has been a shift towards rapid reviews investigating policy questions. Here, the focus is on providing immediate value to addressing the problem at hand. For example, rapid reviews like Evidence Check from the Sax Institute [55, 56] commence with a collaborative process where policymakers and a knowledge broker develop a structured review proposal that describes the policy issue or decision for which the evidence review is required, and articulate the review questions and scope. The process aims to ensure the review will provide policymakers with information specific to their decision and context in a timely way. This collaborative approach has been shown to be well suited for assisting in planned policymaking processes and choosing between specific policy options [55, 57]. The use of knowledge brokers is integral to organising the interactive process between researchers and policymakers so that they can co-produce feasible and research-informed policy options [56].

Policy briefs begin with a policy issue and present evidence to answer the question ‘What should we do?’ A policy brief provides a rationale for choosing a policy alternative or course of action based on the synthesised research findings. They are more practical, flexible and timely in supporting evidence-informed decision-making [49] and can also consider how the evidence fits with prevailing values, beliefs and political context [49]. However, the final product is still a static assessment that is unable to adequately account for changes over time or test the prevailing real-world hypotheses and assumptions [53, 58].

However, participatory dynamic simulation modelling processes go further, providing a platform for explicit synthesis of empirical evidence, local data, expert- and practice-based knowledge, conceptual models and theory to construct, quantify and test a detailed representation of causal factors and the mechanisms of intervention effects [40, 41, 58, 59]. The resulting dynamic model becomes a decision support tool that can step beyond comprehensive approaches, for example, in the prevention of chronic disease, to be used as a ‘what if’ tool to simulate various policy and practice scenarios, and systematically explore the trade-offs of a range of intervention options [41, 58].

### Embedding deliberative methods

An important strategy in knowledge mobilisation theory and practice is the incorporation of deliberative methods. The value of the deliberative process is that it increases understanding of the evidence, and of the competing issues and values, through the engagement and contribution of participants with different perspectives [60]. By deliberating on a problem and its potential solutions, participants strengthen their capacity to address a policy issue and gain confidence in influencing the policy agenda [60].

Processes such as deliberative dialogues involve group interactions that integrate and interpret multiple forms of evidence to inform policy development [61]. Key elements of a deliberative dialogue process include a meeting environment that is conducive to open deliberation about a policy issue, bringing together a mix of participants that ensures fair representation of all relevant interests, and fostering a more equal knowledge base among participants through the presentation of research evidence [60].

Deliberative approaches tend to emphasise the rigour and fairness of the process and try not to anticipate or pre-determine the outcomes of the deliberation. They rely on skilled and neutral facilitation and, while consensus building may be achieved, it is not the primary aim [62]. This requires flexibility and acceptance that the boundaries and scope may be changed as people reflect and discuss the problem, and sometimes modify the questions they want to address.

Thus, translation of research has progressed from managed and controlled dissemination initiatives with pre-defined targets [63]. For knowledge mobilisation, the social, relational and contested nature of true deliberative dialogue or ‘exchange’ relies on negotiated meanings and less predictable outcomes [62, 64, 65].

Participatory dynamic simulation modelling incorporates a deliberative process where stakeholders articulate and develop their understanding of how multiple variables, factors and interventions interact [66, 67], and provides a neutral platform for engaging stakeholders with conflicting views [59]. The participatory model development necessitates in depth deliberation to map a shared mental model of the causal pathways for the focus issue, and the mechanisms by which interventions have an effect on outcomes [68]. The map is then quantified, drawing on research evidence and other data sources through an iterative process of theory testing and building in collaboration with participants. Model outputs are compared with real-world historic data patterns across a range of indicators to establish the validity of the model as an accurate representation of the real-world system. The resulting model becomes a decision support tool that can be used to consider and compare alternative policy options [66, 67]. The model can be refined, updated and customised through ongoing dialogue. Both the process of model development and the results produced by the model enhance stakeholder knowledge and understanding of the system and its dynamics in varying conditions. The process identifies and clarifies complex and contested real world problems [47] and the impact of solutions, and facilitates the implementation of actions based on the available evidence [68, 69].

### Emphasising co-production of knowledge

Co-creation and co-production are two terms used to refer to the process of individuals from different sectors working together to produce an output or outcome such as goods, services or research [70]. Co-production of evidence aims to overcome the often described disconnect between researchers and research end-users, such as health policymakers and programme planners [14]. This concept has been applied to social service design and delivery [71] and increasingly to health research [72–74].

Research translation is embedded in the co-production and partnership approach as the end users are active participants in, and in some cases the drivers for, all phases of the research project [14]. The key elements of co-creation include involving participants as active and equal partners from beginning to end, encouraging reciprocity and sharing of resources and knowledge, and aiming for a ‘transformative’ outcome, i.e. where the research builds capacity and/or has a practical impact on decision-making [14, 71]. The establishment of effective co-production partnerships is an iterative journey, where structures, boundaries and even the purpose of the project are re-negotiated throughout the project dialogue [75].

Relationships and collaborations are routinely identified as key factors in systems approaches [76]. Participatory dynamic modelling provides a structure to facilitate multidisciplinary partnerships, co-learning and co-production. The participatory approach adopts co-production as its driving principle and places the end-user decision-makers at the centre of the process. The decision-makers define the model scope and purpose, and engage multidisciplinary expert stakeholders in the model design and parameterisation (and contribute the identification of data to be used in the model).

Participatory dynamic simulation modelling involves engaging multidisciplinary stakeholders in a deliberative group model-building process where participants discuss evidence and share knowledge about the causal mechanism of the issue being modelled and where and how interventions have their effect within the articulated mechanism. Participatory modelling approaches aim to combine diverse perspectives to tackle the social complexity of problems and recognise that different types of knowledge contribute alternative and valuable perspectives to the problem discourse [47, 59]. The involvement of decision-makers as participants in the model development and validation increases their sense of ownership and confidence that the model is valid for their local context; they are therefore more likely to draw on the model’s outputs to inform decisions about priority interventions and policies [68, 77, 78].

## Reflections on process and early learning from participatory dynamic simulation modelling as a knowledge mobilisation approach

The Australian Prevention Partnership Centre (http://preventioncentre.org.au/), in collaboration with jurisdictional governments, has pioneered the co-production of sophisticated, multiscale dynamic simulation models to support policy and practice. In developing these models, researchers partnered with health departments, clinicians and regional planners in collaboration with a multidisciplinary group of stakeholders using a participatory process [38, 39]. The case studies are described in Box 1.

**Box 1.** Case study descriptions

**Table:** 

*Case Study 1. Model behaviour: A systems approach to reducing alcohol-related harm*
This project was implemented as a collaboration between The Australian Prevention Partnership Centre, the New South Wales Ministry of Health (NSW Health), and local and national alcohol researchers, clinicians and programme planners to inform strategies for reducing alcohol-related harms in NSW.
Alcohol misuse is a complex, systemic problem. Globally, alcohol has been estimated to cause 3.3 million deaths each year, and the costs of alcohol-related harms amount to more than 1% of gross national product in high-income countries. In Australia, alcohol accounts for approximately 3.2% of the total burden of disease and injury, and is estimated to cost AU$15.3 billion each year [79, 80].
The design of effective responses to this problem has been challenged by a lack of clarity on the mechanisms driving alcohol misuse and its associated harms, differing views of stakeholders regarding the most appropriate and effective intervention approaches, a lack of evidence supporting commonly implemented and acceptable intervention approaches, and strong evidence for less acceptable interventions. As a consequence, political considerations, community advocacy and industry lobbying contribute to a hotly contested debate on what is the most appropriate course of action.
The developed model uniquely captures the heterogeneity of drinking behaviours across the NSW population, the dynamics of those drinking behaviours across the life course, the acute and chronic harms that arise from those behaviours, and the differential effects of interventions across subgroups in the population. Testing of the model demonstrated its ability to reproduce a range of real world data patterns, which provides confidence that the model can produce robust forecasts of the comparative impacts of interventions into the future. The model is currently being used to engage with broader policy stakeholders to demonstrate the value of such models in informing effective and acceptable strategies for reducing alcohol-related harms [38].
*Case Study 2. Premier’s Priority Project – reducing childhood overweight and obesity by 5%*
In September 2015, the NSW Premier unveiled 30 State priorities to grow the economy, deliver infrastructure, protect the vulnerable and improve health, education and public services across NSW. Included in these areas of focus were the 12 Premier’s Priorities, including an ambitious target to reduce childhood overweight and obesity in children by 5% over 10 years.
Based on population projections and the anticipated impact of enhancing the existing suite of interventions delivered by NSW Health, it was estimated that additional strategies, or combinations of strategies, would be required to achieve the Premier’s target. However, the complexity of the problem and uncertainty about where best to target resources and efforts presented a challenge to decision-makers. To address this, the Australian Prevention Partnership Centre in partnership with NSW Health undertook to co-develop a system dynamics model of childhood overweight and obesity.
The model development process engaged a broad range of multidisciplinary stakeholders working in the area of childhood obesity spanning the fields of academia, service delivery, policy, planning and infrastructure. Through a series of participatory workshops the problem was collaboratively mapped and interventions to be included in the model prioritised. The map was conceptualised as a computational model, quantified, tested and validated against historic data, and iteratively refined through feedback sought during and between workshops.
The model is being used by NSW Health and their stakeholders to test the likely impacts of a range of policies and programmes, and to inform the combination of interventions that might achieve the Premier’s target.
*Case Study 3. Simulation modelling for Diabetes in Pregnancy (DIP) in the Australian Capital Territory (ACT)*
This project was implemented as a collaboration between The Australian Prevention Partnership Centre, ACT Health Directorate (ACT Health), local and national researchers, clinicians and policymakers. DIP is increasing both in the ACT and Australia, and diabetes services are having difficulty meeting demand with existing resources. The increase in DIP is associated with increasing prevalence of risk factors such as overweight and obesity, older maternal age and increasing numbers of women from high-risk ethnic groups. Changes to diagnostic screening has resulted in women being diagnosed with DIP earlier in their pregnancy and therefore requiring services for a longer period of time. Women are also more frequently presenting with a number of risk factors resulting in more complex care needs.
A dynamic simulation model focusing on DIP from an ACT perspective was developed. The national context was considered in the model development, with the model being considered a proof of concept with the potential to expand more broadly.
The model considers the short, intermediate and long-term implications of the increasing prevalence of risk factors for DIP. Prevention of risk factors was prioritised in the model as small delays in the development of diabetes will have large implications for the longer term burden of disease and costs to the health system.
Alternative models of care for DIP were considered in the model. The rising prevalence of DIP is having a significant impact on health service demand and resources, and the need to ‘do things differently’ was identified by participants. The model informs the investments for intervention in DIP, including both clinical and population health interventions. Workload and resource use has been incorporated into the model to enable it to act as a resource allocation decision support tool. At the time of publication, this model was being finalised.

The participatory simulation modelling processes and activities utilised in these case studies have been described in detail elsewhere [38, 39]. However, a brief overview of the process and examples of activities are provided in Box 2 to give context for the discussion below.

**Box 2.** Overview of the process and examples of activities

**Table:** 

**Project planning and engagement (Fig.** 1 **)**
Early engagement with stakeholders for each case study was undertaken to identify a priority problem, and determine and define policy priorities requiring decision support methods. A domain expert, preferably from the primary partner organisation (partner), was identified to be a lead collaborator in the project (lead domain expert). This role included supporting the engagement of stakeholders and co-facilitating workshops.
Project planning meetings were held to clearly define the aspects of the problem to be modelled and its scope and boundaries, as well as to identify key outputs of interest and intervention options to be included and tested by the model.
Experts and key participants with an important ‘stake’ in the topic were identified and invited to participate in the model development group (participants). Group composition was purposefully considered to ensure inclusion of a diverse range of views and identification of participants who were considered reliable and reputable representatives of broader stakeholder groups (stakeholders). Background reading material regarding simulation modelling and the topic of interest was sent to participants prior to the workshop to provide a platform of common understanding.
**Model building and validation**
Through a series of participatory workshops, the model building group, informed by collated evidence and data, collaboratively identified and mapped the key risk factors and likely causal pathways leading to outcomes of interest for the focus topic of the model.
The proposed model architecture was presented at the first workshop, and then subsequent versions of the model were developed to reflect participant language, input and feedback as well as providing increased detail and maturity.
Participants were familiarised with the model infrastructure using paper-based physical representations. For example, during one activity, participants built a physical representation of the model, with model components represented in card and tape. Participants worked collaboratively to document factors that contribute to the problem being modelled and mapped these directly onto the card and tape representation (Fig. 2).
Similar activities were conducted to involve participants in mapping the mechanisms through which interventions would impact the model (Fig. 3).
The interim conceptual map or model was tested and validated in collaboration with the model building group during each workshop.
The workshop structure was flexible to account for differences in group size and incorporated a range of activities with the whole group or smaller sub-groups as appropriate to allow participants to raise issues, negotiate perspectives and build consensus. For activities where the group was split, the modelling team allocated participants to ensure each sub-group included a range of perspectives and areas of expertise, and to encourage productive group dynamics.
**Consensus building for policy actions**
Final half-day workshops and follow-up webinars were conducted where the model was presented back to the model building group for verification, discussion, consensus, feedback of results and further input on preferred visualisation of model outputs.
Outputs from modelled scenarios were presented to participants to facilitate the development of new insights and knowledge about the likely impact of interventions and discussion about potential policy actions.
Examples of the user interface and model outputs are presented in Figs. 4 and 5. These figures illustrate how model users create scenarios to test and compare the outcomes for different combinations of selected interventions. Figure 4 includes the user interfaces from the Alcohol-related harm (top image) and the Premier’s Priority (bottom image) case studies.
The model outputs take the form of dynamic visualisations and graphs that represent model outcomes for created scenarios, e.g. for variations of intervention effectiveness and reach. These can be compared against benchmark or ‘business as usual’ model outputs. Figure 5 presents example outputs from the Alcohol-related harm case study. 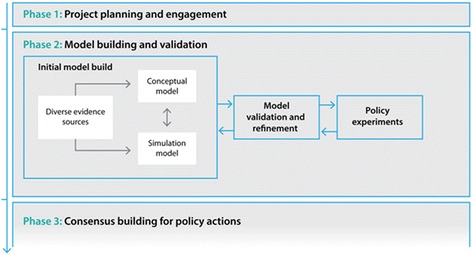 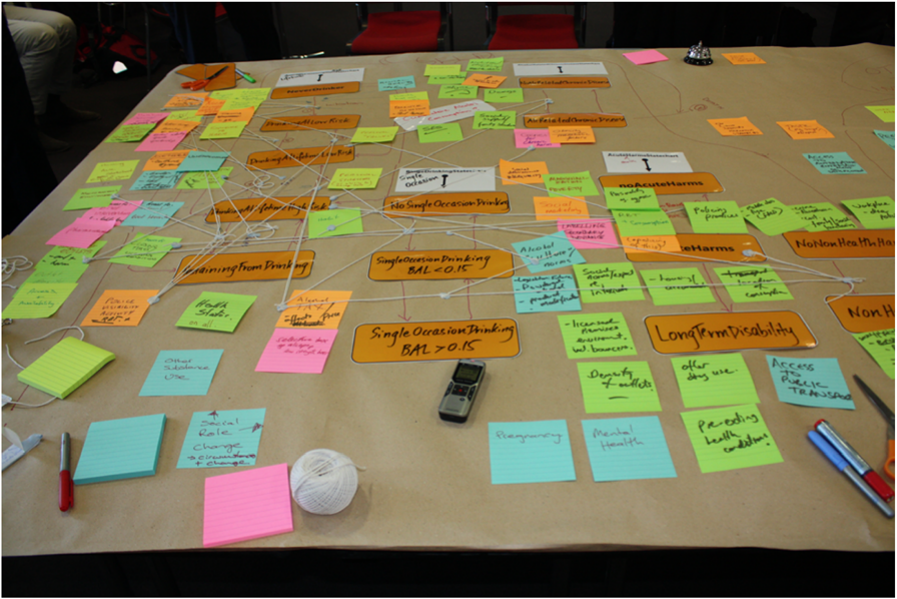 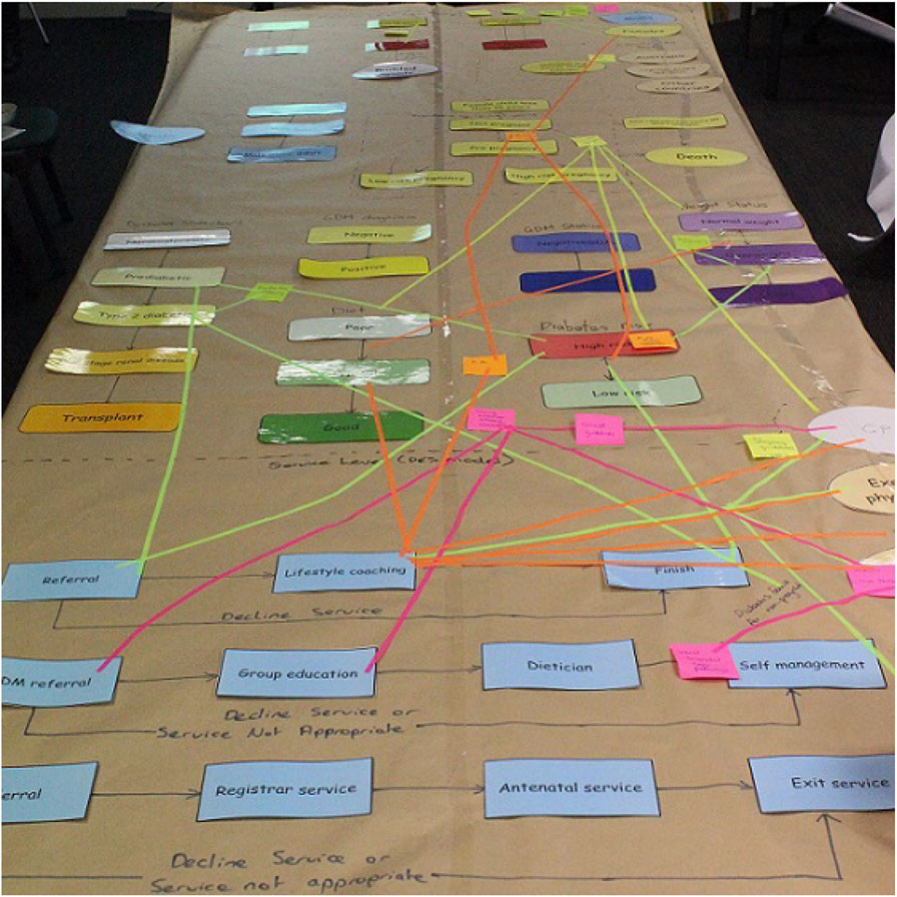 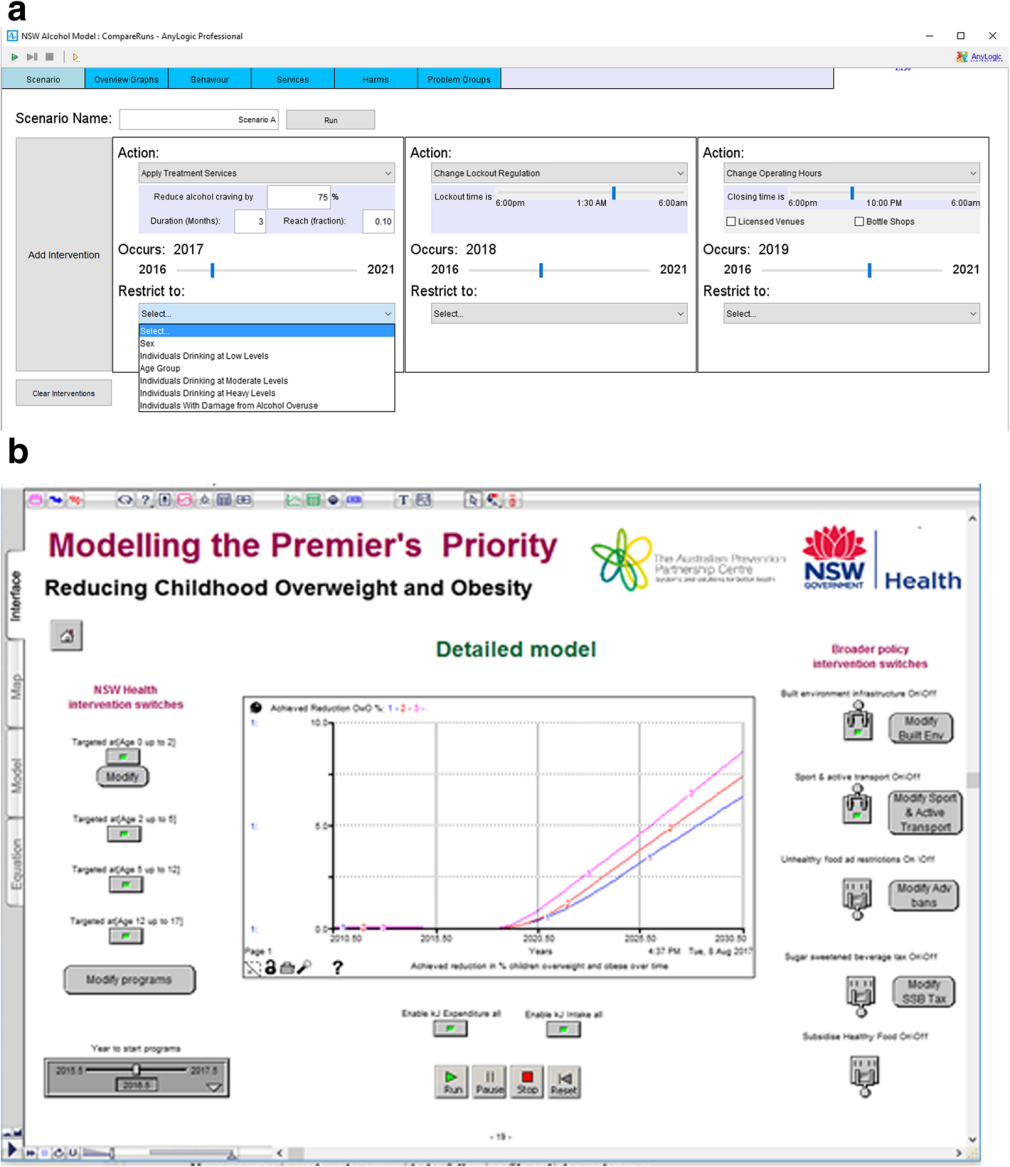 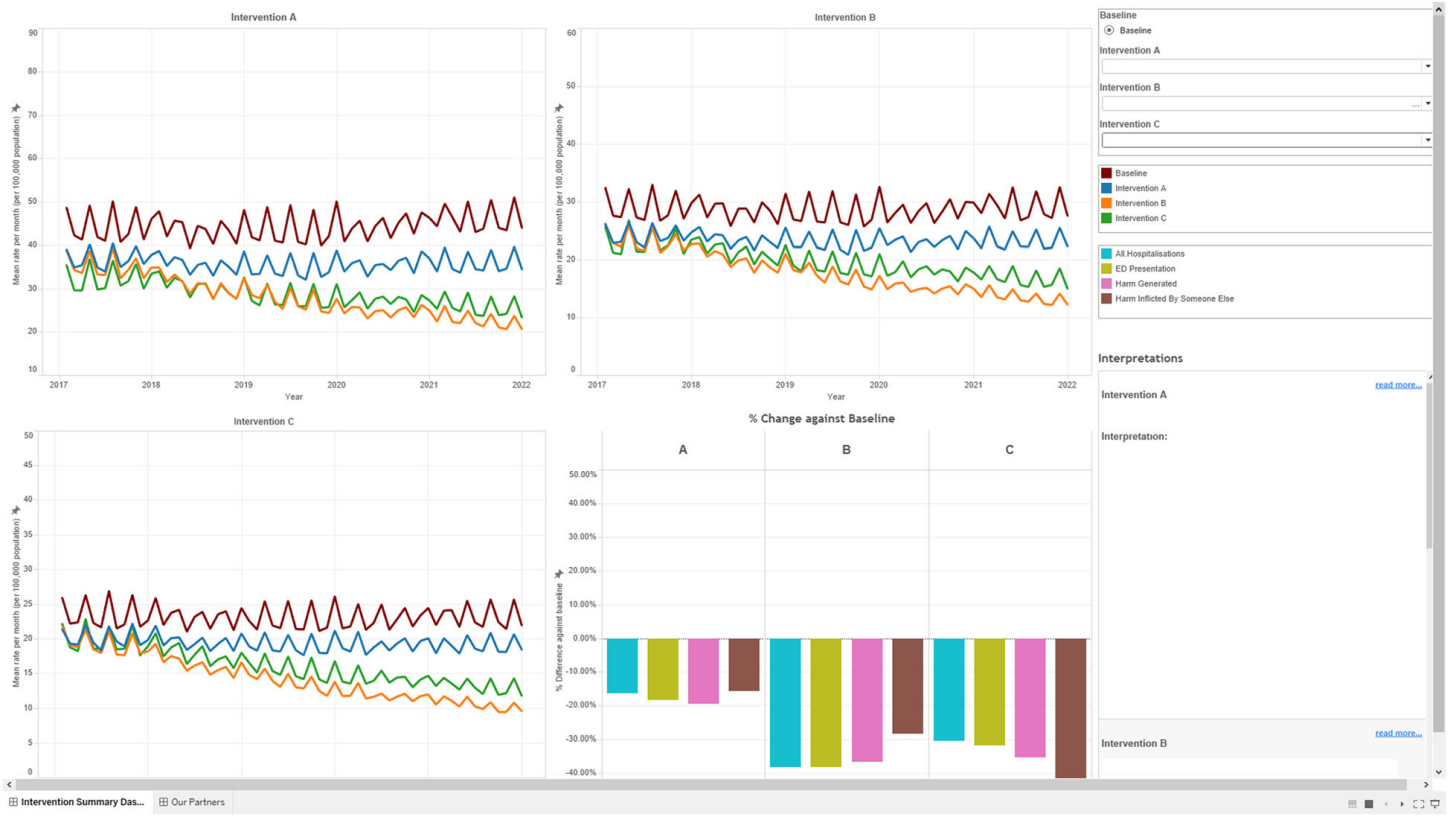

## Key aspects of operationalising participatory dynamic simulation modelling

In the remainder of this paper we draw on our experience of operationalising participatory dynamic simulation modelling to support chronic disease prevention policy and practice in Australia. We consider three key aspects of the process, (1) establishing partnerships with stakeholders, (2) engaging participants actively in the modelling process and (3) using co-production methods to build trust in the model and its outputs. We then discuss some of the lessons and implications for adopting these approaches in contemporary knowledge mobilisation practice.

### Establishing effective partnerships with stakeholders

In each of the case studies, the focus of the model was proposed by Australian Capital Territory Health or New South Wales Health as a priority area with current local concern, complex causal risk factors and as issues where previous policy responses had limited impact. The health jurisdictions were therefore coming to the process with a view that they needed to do things differently and were motivated to work on innovative solutions.

Engagement with these primary partner organisations (partners) continued throughout the modelling process, from identifying relevant subject matter experts to be involved (participants), to soliciting input on relevant data and literature sources, negotiating the model purpose, scope and structure, and encouraging involvement in the facilitation of modelling workshops.

Identifying and including a lead domain expert for each case study, e.g. a public health practitioner or clinician, who was well respected and associated with the partner, increased engagement, solidified the partnerships and built trust in the modelling process. These lead domain experts acted as co-facilitators for model development workshops, along with the project leader from the modelling team. The combination of domain and modelling expertise allowed workshop co-facilitators to navigate interdisciplinary participation through a process of developing a common language and understanding to facilitate model development.

### Engaging participants actively in the modelling process

Facilitated workshop activities were designed to involve participants actively in the modelling process (examples in Box 2). These methods supported participant engagement and investment in the model as they deliberated and negotiated with each other to prioritise causal factors, their interactions, and interventions and outcomes to be captured in the model. Significant learning occurred through these deliberative dialogues, with participants reporting that their ‘interaction was key’ to the modelling process. The mapping activities provided an interactive opportunity for participants to synthesise their collective knowledge and expertise with quantitative evidence.

The practical hands-on mapping activities used during workshops (Box 2) also familiarised participants with the model architecture. The model architecture (the diagrammatic representation of the computer model) physically represented how the identified causal factors, interventions and resulting outputs were incorporated into the model. This allowed for two-way learning as increased familiarity and confidence in understanding the model architecture enabled participants to critique and provide feedback to modellers to ensure the model accurately represented their shared understanding. Taking an iterative approach facilitated collective learning, and the demonstration of the evolving model at the second and subsequent workshops further validated and improved model design.

At times, the priorities of policy partners (in terms of interventions and outputs to be included in the model) differed from those of subject matter experts. The participatory process of negotiation helped to build consensus on what to prioritise in the model and enhanced each participant group’s understanding of the others’ knowledge and research or policy. Expert facilitation skills were necessary to draw out diverse contributions, maintain engagement in the process and negotiate compromises where necessary. Explicit processes, including voting, were used to democratically resolve disagreements and to clarify priorities in model development.

### Co-production built trust in model outputs and facilitated consensus for action

The strong partnerships and active engagement of partners and participants throughout the iterative model development were critical for building trust in model outputs and providing the best opportunity for impact on policy and programme decisions.

The use of co-production methods as described above increased transparency in the model building process. Demonstrating the model conceptualisation to participants at each workshop and highlighting their contributions increased participants’ understanding of the model. This transparency reinforced the value of their participation and their ownership of the model, and provided an opportunity to establish the face validity of models against expert and local knowledge.

Another important aspect to building trust in model outputs was to encourage discussion about the limitations and assumptions in the model design and available data sources. Documentation of data sources and assumptions built into the models was shared with participants to critically evaluate and provide feedback.

Building participants’ trust in the model was necessary for its acceptance by stakeholders/experts who were not involved in its development (stakeholders). Involvement of key opinion leaders in the model development groups brought credibility to the models as participants acted as ambassadors for the model within their broader stakeholder groups. Diversity of expertise within the participant group was also important so different stakeholder groups felt their perspective had been represented.

When some model outputs did not confirm long held beliefs about likely effects of interventions and their combinations, there was robust debate about the implications and caution in using such results to inform decision-making. In these situations, it was particularly useful to invite stakeholders to interact with the model, challenge their assumptions, provide alternative data and test their expectations against model outputs. Iterative, open and non-defensive communication was critical to facilitating these interactions, advancing understanding of the complex problem and building trust in the decision support tool.

Model validation is an essential stage of all model development, including models developed using participatory approaches [77]. Demonstrating to partners, participants and stakeholders that the models reproduced historic data patterns across a range of indicators confirmed their validity, and built confidence that the models would produce robust forecasts into the future.

Collaborative processes were also used to maximise the potential usability of the model. Participant engagement with the model was encouraged to test and refine the user interface. This user testing ensured that the interface was intuitive and accessible for a diverse range of users.

## Conclusion

The participatory dynamic simulation modelling processes utilised in these case studies built on knowledge mobilisation best practice and produced dynamic decision support tools that integrated diverse forms of evidence, including research evidence, expert knowledge and localised contextual information. The participatory approach placed end-users at the centre of the process and embedded deliberative methods and co-production of knowledge. Policymakers, researchers, scientists, clinicians, consumers and modellers collaborated and explored policy and health service scenarios for priority public health topics.

An important element of co-production in these case studies was equal partnering with key stakeholders to negotiate the priority issue to be modelled. These were ‘hot topics’ that were current, locally relevant, had complex causal mechanisms, and for which decision-makers needed to decide between competing courses of action. It was in these circumstances that participatory dynamic modelling provided an opportunity for policy and programme options and combinations to be tested within a safe, simulated environment before being implemented in the real world. The case studies revealed valuable lessons for the participatory dynamic simulation modelling process in health policy settings. The case study topics were complex and multi-faceted, and the diverse representation of stakeholders in the modelling groups was essential as no one individual could be an expert on all aspects of the issue. An aspect of the process was thus to emphasise the need for knowledge sharing among stakeholders and to develop a common understanding of the issue, and of the potential interventions to address it. Differential participation did occur in some of the workshops, e.g. participants sometimes deferred to those they perceived to have greater authority or expertise for particular aspects of the content. However, the workshop facilitators promoted the value of diverse perspectives in building a robust model and regularly sought to draw out those who were less vocal.

Consistent with other knowledge mobilisation approaches, the participatory process was time consuming and required ongoing efforts to maintain and coordinate diverse engagements. However, the challenges were outweighed by the positive outcomes of effective collaborative networks, co-production of knowledge, and capacity to integrate diverse evidence and expert opinion. In our case study settings, many participants had limited or no prior experience with dynamic simulation models or the modelling process. An important dimension of the knowledge mobilisation process was the translation that occurred between disciplines, e.g. clinicians, computer scientists and population health professionals, to ensure that everyone understood each other’s perspectives and were working toward a common goal.

The processes of data gathering and synthesis commonly highlighted gaps in local programme outcome data, as well as in the published literature. For example, locally available evaluation data was frequently limited to process and participation measures, rather than programme outcomes and effectiveness. Often, the local contextually relevant data was utilised and triangulated with other potentially more reliable but less locally relevant sources to inform the models. Whilst evidence gaps are an ongoing challenge in all settings, the process of uncovering these gaps through the participatory process, and prioritising data needs through sensitivity testing in the model, provided important information for prioritising future research and guiding refinement of local programme evaluations and routine data collection.

An important challenge of knowledge mobilisation using this participatory modelling approach was the building of trust in the model outputs. This was less of an issue when the model outputs confirmed existing preconceptions of underlying causal mechanisms, but significantly more challenging when model outputs were contrary to participants’ long held beliefs. This could be particularly challenging for subject matter experts who were required to reassess their prior expectations. However, the dynamic, interactive nature of the models as decision support tools facilitated ongoing dialogue and negotiation with stakeholders and developed understanding and trust.

Our analysis of participatory model building methods is ongoing. Further activities in this programme of research involve evaluating the perceived value of the participatory process; the commitment and confidence of partners and participants to implement policy and programme decisions identified through the modelling process; and the impact of the process, i.e. how model outputs will be used to inform policy and programme decisions in the local public health settings.
